# Variational Online Learning of Neural Dynamics

**DOI:** 10.3389/fncom.2020.00071

**Published:** 2020-10-14

**Authors:** Yuan Zhao, Il Memming Park

**Affiliations:** ^1^Department of Neurobiology and Behavior, Stony Brook University, Stony Brook, NY, United States; ^2^Center for Neural Circuit Dynamics, Stony Brook University, Stony Brook, NY, United States; ^3^Institute for Advanced Computational Science, Stony Brook University, Stony Brook, NY, United States

**Keywords:** state space models, online learning, variational Bayes, filtering, system identification

## Abstract

New technologies for recording the activity of large neural populations during complex behavior provide exciting opportunities for investigating the neural computations that underlie perception, cognition, and decision-making. Non-linear state space models provide an interpretable signal processing framework by combining an intuitive dynamical system with a probabilistic observation model, which can provide insights into neural dynamics, neural computation, and development of neural prosthetics and treatment through feedback control. This brings with it the challenge of learning both latent neural state and the underlying dynamical system because neither are known for neural systems *a priori*. We developed a flexible online learning framework for latent non-linear state dynamics and filtered latent states. Using the stochastic gradient variational Bayes approach, our method jointly optimizes the parameters of the non-linear dynamical system, the observation model, and the black-box recognition model. Unlike previous approaches, our framework can incorporate non-trivial distributions of observation noise and has constant time and space complexity. These features make our approach amenable to real-time applications and the potential to automate analysis and experimental design in ways that testably track and modify behavior using stimuli designed to influence learning.

## 1. Introduction

Discovering interpretable structure from a streaming high-dimensional time series has many applications in science and engineering. Since the invention of the celebrated Kalman filter, state space models have been successful in providing a succinct (and thus a more interpretable) description of the underlying dynamics that explains the observed time series as trajectories in a low-dimensional state space. Taking a step further, state space models equipped with non-linear dynamics provide an opportunity to describe the latent “laws” of the system that is generating the seemingly entangled time series (Haykin and Principe, 1998; Ko and Fox, 2009; Mattos et al., 2016). Specifically, we are concerned with the problem of identifying a continuous non-linear dynamics in the state space **x**(*t*) ∈ ℝ^*d*^ that captures the spatiotemporal structure of a noisy observation **y**(*t*):

(1a) $$\begin{array}{ll} \dot{\text{x}}=F_{\theta }\left(\text{x}\left(t\right),\text{u}\left(t\right)\right) & \text{(state dynamics)} \end{array}$$

(1b) $$\begin{array}{ll} \text{y}\left(t\right)\sim P\left(\text{y}\left(t\right)|G_{\theta }\left(\text{x}\left(t\right),\text{u}\left(t\right)\right)\right) & \text{(observation model)} \end{array}$$

where *F* and *G* are continuous functions that may depend on parameter θ, **u**(*t*) is the control input, and *P* denotes a probability distribution that captures the noise in the observation, e.g., Gaussian distribution for field potentials or Poisson distribution for spike counts.

In practice, the continuous-time state dynamics is more conveniently formulated in discrete time as

(2) $$\begin{array}{ll} {\text{x}}_{t+1}=f_{\theta }\left({\text{x}}_{t},{\text{u}}_{t}\right)+{\text{ϵ}}_{t} & \text{(discrete time state dynamics)} \end{array}$$

where **ϵ**_*t*_ is intended to capture the unobserved (latent) perturbations of the state **x**_*t*_. Such (spatially) continuous state space models are natural in many applications where the changes are slow and the underlying system follows physical laws and constraints (e.g., object tracking) or where learning the laws are of great interest (e.g., in neuroscience and robotics) (Roweis and Ghahramani, 2001; Mante et al., 2013; Sussillo and Barak, 2013; Frigola et al., 2014; Zhao and Park, 2017). Specifically, in the context of neuroscience, the state vector **x**_*t*_ represents the instantaneous state of the neural population, while *f* captures the time evolution of the population state. Further interpretation of *f* can provide understanding as to how neural computation is implemented (Mante et al., 2013; Zhao and Park, 2016; Russo et al., 2018).

If the non-linear state space model is fully specified, Bayesian inference methods can be employed to estimate the current state (Ho and Lee, 1964; Särkkä, 2013). Conventionally, the estimation of latent states using only the past observation is referred to as filtering—inference of the filtering distribution, *p*(**x**_*t*_∣**y**_≤*t*_). If both past and future observations are used, then the quantity of interest is usually the smoothing distribution, *p*(**x**_≤*t*_∣**y**_≤*t*_)). We are also interested in predicting the distribution over future states, *p*(**x**_*t*:*t*+*s*_∣**y**_≤*t*_), and observations, *p*(**y**_*t*+1:*t*+*s*_∣**y**_≤*t*_) for *s* > 0. In many applications, however, the challenge is in learning the parameters θ of the state space model (a.k.a. the system identification problem). We aim to provide a method for simultaneously learning both the latent trajectory **x**_*t*_ and the latent (non-linear) dynamical and observational system θ, known as the *joint estimation problem* (Haykin, 2001).

Expectation maximization (EM) based methods have been widely used in practice (Ghahramani and Roweis, 1999; Valpola and Karhunen, 2002; Turner et al., 2010; Golub et al., 2013), and more recently variational autoencoder methods (Archer et al., 2015; Krishnan et al., 2015, 2016; Watter et al., 2015; Johnson et al., 2016; Karl et al., 2017) have been proposed, all of which are designed for offline analysis and are not appropriate for real-time applications. Recursive stochastic variational inference has been successful in streaming data assuming independent samples (Broderick et al., 2013), however, in the presence of temporal dependence, proposed variational algorithms (e.g., Frigola et al., 2014) remain theoretical and lack testing.

In this study, we are interested in *real-time* signal processing and state space control setting (Golub et al., 2013) where online algorithms are needed that can recursively solve the joint estimation problem on streaming observations. A popular solution to this problem exploits the fact that online state estimators for non-linear state space models such as extended Kalman filter (EKF) or unscented Kalman filter (UKF) can be used for non-linear regression formulated as a state space model. By augmenting the state space with the parameters, one can build an online *dual* estimator using non-linear Kalman filtering (Wan and Van Der Merwe, 2000; Wan and Nelson, 2001). They involve, however, coarse approximation of Bayesian filtering and many hyperparameters, do not take advantage of modern stochastic gradient optimization, and are not easily applicable to arbitrary observation likelihoods. There are also closely related online version of EM-type algorithms (Roweis and Ghahramani, 2001) that share similar concerns.

In hopes of lifting these concerns, we derive an *online* black-box variational inference framework, referred to as variational joint filtering (VJF), applicable to a wide range of non-linear state dynamics (dynamic models) and observation models, that is, the computational demand of the algorithm is constant per time step. Our approach is aimed as follows:

1. **Online adaptive learning**: Our target application scenarios are streaming data. This allows the inference during an experiment or as part of a neural prosthetics. If the system changes, the inference will catch up with the altered system parameters.
2. **Joint estimation**: The proposed method is supposed to simultaneously learn the latent states *p*(**x**_*t*_∣**y**_≤*t*_), state dynamics *f*(**x**_*t*_, **u**_*t*_) and the observation model *G*(**x**, **u**). No offline training is necessary to learn the system parameters.
3. **Interpretability**: Under the framework of state space modeling, rather than interpret the system via model parameters, we employ the language of dynamical systems and capture the characteristics of the system qualitatively via fixed point, limit cycle, strange attractor, bifurcation, and so on, which are key components of theories of neural dynamics and computation.

We focus on low-dimensional latent dynamics that often underlie many neuroscientific experiments and allow for producing interpretable visualizations of complex collective network dynamics in this study.

## 2. Variational Principle for Online Joint Estimation

The crux of recursive Bayesian filtering is updating the posterior over the latent state one step at a time:

(3) $$\begin{array}{l} p\left({\text{x}}_{t}|{\text{y}}_{\leq t}\right)=\underset{\text{likelihood}}{\underset{︸}{p\left({\text{y}}_{t}|{\text{x}}_{t}\right)}}\underset{\text{prior at time }t}{\underset{︸}{p\left({\text{x}}_{t}|{\text{y}}_{<t}\right)}}\;/\underset{\begin{array}{c} \text{marginal likelihood} \end{array}}{\underset{︸}{p\left({\text{y}}_{t}|{\text{y}}_{<t}\right)}} \end{array}$$

where the input **u**_*t*_ and parameters θ are omitted for brevity. Unfortunately, the exact calculations of Equation (3) are not tractable in general, especially for non-linear dynamic models and/or non-conjugate distributions. We thus turn to approximate inference and develop a recursive variational Bayesian filter by deriving an evidence lower bound for the marginal likelihood as the objective function. Let *q*(**x**_*t*_) denote an arbitrary probability measure that will eventually approximate the filtering density *p*(**x**_*t*_∣**y**_≤*t*_). From Equation (3), we can rearrange the marginal log-likelihood as

$$\begin{array}{l} \log p\left({\text{y}}_{t}|{\text{y}}_{<t}\right)\\ =\log\frac{p\left({\text{y}}_{t}|{\text{x}}_{t}\right)p\left({\text{x}}_{t}|{\text{y}}_{<t}\right)}{p\left({\text{x}}_{t}|{\text{y}}_{\leq t}\right)}\;\text{for any }{\text{x}}_{t}\\ ={𝔼}_{q\left({\text{x}}_{t}\right)}\left[\log\frac{p\left({\text{y}}_{t}|{\text{x}}_{t}\right)p\left({\text{x}}_{t}|{\text{y}}_{<t}\right)}{p\left({\text{x}}_{t}|{\text{y}}_{\leq t}\right)}\right]\;\text{the marginal is constant to }q\left({\text{x}}_{t}\right)\\ ={𝔼}_{q\left({\text{x}}_{t}\right)}\left[\log\frac{p\left({\text{y}}_{t}|{\text{x}}_{t}\right)p\left({\text{x}}_{t}|{\text{y}}_{<t}\right)q\left({\text{x}}_{t}\right)}{p\left({\text{x}}_{t}|{\text{y}}_{\leq t}\right)q\left({\text{x}}_{t}\right)}\right]\\ =\underset{\begin{array}{c} \text{reconstruction log-likelihood} \end{array}}{\underset{︸}{{𝔼}_{q\left({\text{x}}_{t}\right)}\left[\log p\left({\text{y}}_{t}|{\text{x}}_{t}\right)\right]}}-{𝔻}_{\text{KL}}\left(q\left({\text{x}}_{t}\right)||p\left({\text{x}}_{t}|{\text{y}}_{<t}\right)\right)\\ +\underset{\begin{array}{c} \text{variational gap \#1} \end{array}}{\underset{︸}{{𝔻}_{\text{KL}}\left(q\left({\text{x}}_{t}\right)||p\left({\text{x}}_{t}|{\text{y}}_{\leq t}\right)\right)}}\\ \geq {𝔼}_{q\left({\text{x}}_{t}\right)}\left[\log p\left({\text{y}}_{t}|{\text{x}}_{t}\right)\right]+\underset{\begin{array}{c} \text{entropy} \end{array}}{\underset{︸}{ℍ\left(q\left({\text{x}}_{t}\right)\right)}}+{𝔼}_{q\left({\text{x}}_{t}\right)}\left[\log p\left({\text{x}}_{t}|{\text{y}}_{<t}\right)\right]\\ ={𝔼}_{q\left({\text{x}}_{t}\right)}\left[\log p\left({\text{y}}_{t}|{\text{x}}_{t}\right)\right]+ℍ\left(q\left({\text{x}}_{t}\right)\right)\\ \;+{𝔼}_{q\left({\text{x}}_{t}\right)}\left[\log{𝔼}_{p\left({\text{x}}_{t-1}|{\text{y}}_{<t}\right)}\left[p\left({\text{x}}_{t}|{\text{x}}_{t-1}\right)\right]\right]\\ ={𝔼}_{q\left({\text{x}}_{t}\right)}\left[\log p\left({\text{y}}_{t}|{\text{x}}_{t}\right)\right]+ℍ\left(q\left({\text{x}}_{t}\right)\right)\\ \;+{𝔼}_{q\left({\text{x}}_{t}\right)}\left[{𝔼}_{p\left({\text{x}}_{t-1}|{\text{y}}_{<t}\right)}\left[\log p\left({\text{x}}_{t}|{\text{x}}_{t-1}\right)\right]\right]\\ \;+\underset{\begin{array}{c} \text{variational gap \#2} \end{array}}{\underset{︸}{{𝔼}_{q\left({\text{x}}_{t}\right)}\left[{𝔻}_{\text{KL}}\left(p\left({\text{x}}_{t-1}|{\text{y}}_{<t}\right)||p\left({\text{x}}_{t-1}|{\text{x}}_{t},{\text{y}}_{<t}\right)\right)\right]}}\\ \geq {𝔼}_{q\left({\text{x}}_{t}\right)}\left[\log p\left({\text{y}}_{t}|{\text{x}}_{t}\right)\right]+ℍ\left(q\left({\text{x}}_{t}\right)\right)\\ \;+{𝔼}_{q\left({\text{x}}_{t}\right)}{𝔼}_{p\left({\text{x}}_{t-1}|{\text{y}}_{<t}\right)}\left[\log p\left({\text{x}}_{t}|{\text{x}}_{t-1}\right)\right] \end{array}$$

where ℍ denotes Shannon's entropy and *D*_KL_ denotes the Kullback-Leibler (KL) divergence (Cover and Thomas, 1991). Maximizing this lower bound would result in a variational posterior *q*(**x**_*t*_) ≈ *p*(**x**_*t*_∣**y**_≤*t*_) w.r.t. *q*(**x**_*t*_). Naturally we plug in the previous step's solution to the next time step, obtaining a loss function suitable for recursive estimation:

(4) $$\begin{array}{l} L:={𝔼}_{q\left({\text{x}}_{t}\right)}\left[\log p\left({\text{y}}_{t}|{\text{x}}_{t}\right)\right]+ℍ\left(q\left({\text{x}}_{t}\right)\right)\\ \;+\underset{\begin{array}{c} \text{dynamics log-likelihood} \end{array}}{\underset{︸}{{𝔼}_{q\left({\text{x}}_{t}\right)}{𝔼}_{q\left({\text{x}}_{t-1}\right)}\left[\log p\left({\text{x}}_{t}|{\text{x}}_{t-1}\right)\right]}} \end{array}$$

This also results in consistent *q*(**x**_*t*_) for all time steps as they are in the same family of distribution.

Meanwhile, as it is aimed to jointly estimate the observation model *p*(**y**_*t*_∣**x**_*t*_) and state dynamics *p*(**x**_*t*_∣**x**_*t*−1_), we achieve online inference by maximizing this objective L w.r.t., their parameters (omitted for brevity), and the variational posterior distribution *q*(**x**_*t*_) simultaneously, provided that *q*(**x**_*t*−1_) takes some parameterized form and has been estimated from the previous time step. Maximizing the objective L is equivalent to minimizing the two variational gaps: (1) the variational filtering posterior must be close to the true filtering posterior, and (2) the filtering posterior from the previous step needs to be close to *p*(**x**_*t*−1_∣**x**_*t*_, **y**_<*t*_). Note that the second gap is invariant to *q*(**x**_*t*_) if *p*(**x**_*t*−1_∣**x**_*t*_, **y**_<*t*_) = *p*(**x**_*t*−1_∣**y**_<*t*_), that is, the one-step backward smoothing distribution is identical to the filtering distribution.

On the flip side, intuitively, there are three components in L that are jointly optimized: (1) reconstruction log-likelihood, which is maximized if *q*(**x**_*t*_) concentrates around the maximum likelihood estimate given only **y**_*t*_, (2) the dynamics log-likelihood, which is maximized if *q*(**x**_*t*_) concentrates at around the maximum of 𝔼_*q*(**x**_*t*−1_)_[log *p*(**x**_*t*_∣**x**_*t*−1_)], and (3) the entropy, which expands *q*(**x**_*t*_) and prevents it from collapsing to a point mass.

In order for this recursive estimation to be real-time, we choose *q*(**x**_*t*_) to be a multivariate normal with diagonal covariance $N\left({\text{μ}}_{t},{\text{s}}_{t}\right)$ where **μ**_*t*_ is the mean vector and **s**_*t*_ is the diagonal of the covariance matrix in this study. Moreover, to amortize the computational cost of optimization to obtain the best *q*(**x**_*t*_) on each time step, we employ the variational autoencoder architecture (Hinton et al., 1995) to parameterize *q*(**x**_*t*_) with a recognition model. Intuitively, the recognition model embodies the optimization process of finding *q*(**x**_*t*_), that is, it performs an approximate Bayesian filtering computation (in constant time) of Equation (3) according to the objective function L. We use a recursive recognition model that maps *q*(**x**_*t*−1_) and **y**_*t*_ to *q*(**x**_*t*_). In particular, the recognition model takes a deterministic recursive form:

(5) $$\begin{array}{l} \left[{\text{μ}}_{t},{\text{s}}_{t}\right]=h\left({\text{y}}_{t},{\text{u}}_{t-1},{\text{μ}}_{t-1},{\text{s}}_{t-1}\right) \end{array}$$

Specifically *h* takes a simple the form of the multi-layer perceptron (MLP) (Hastie et al., 2009) in this study, and we refer to its parameters as the recognition model parameters. Note that the recursive architecture of the recognition model reflects the Markovian structure of the assumed dynamics (c.f., smoothing networks often use bidirectional recurrent neural network (RNN) (Sussillo et al., 2016) or graphical models (Archer et al., 2015; Johnson et al., 2016)).

The expectations appearing in the reconstruction log-likelihood and dynamics log-likelihood are not always tractable in general. For those intractable cases, one can use the reparameterization trick and stochastic variational Bayes (Kingma and Welling, 2014; Rezende et al., 2014): rewriting the expectations over *q* as expectation over a standard normal random variable, i.e., ${\text{μ}}_{t}+{\text{s}}_{t}^{\frac{1}{2}}N\left(0,1\right)$, and using a single sample for each time step. Hence, in practice, we optimize the following objective function (the other variables and parameters are omitted for brevity),

(6) $$\begin{array}{l} \hat{L}=\log p\left({\text{y}}_{t}|{\tilde{\text{x}}}_{t},\theta \right)+{𝔼}_{q\left({\text{x}}_{t}\right)}\log p\left({\text{x}}_{t}|{\tilde{\text{x}}}_{t-1},\theta \right)+H\left(q\left({\text{x}}_{t}\right)\right) \end{array}$$

where ${\tilde{\text{x}}}_{t}$ and ${\tilde{\text{x}}}_{t-1}$ represent random samples from *q*(**x**_*t*_) and *q*(**x**_*t*−1_) respectively. Note that the remaining expectation over *q*(**x**_*t*_) has a closed form solution under our Gaussian state noise, **ϵ**_*t*_, assumption. Our method can thus handle arbitrary observation and dynamic models, unlike dual form non-linear Kalman filtering methods, which usually suffer difficulties in sampling, e.g., transforming Gaussian random numbers into point process observations.

**Figure 1:**
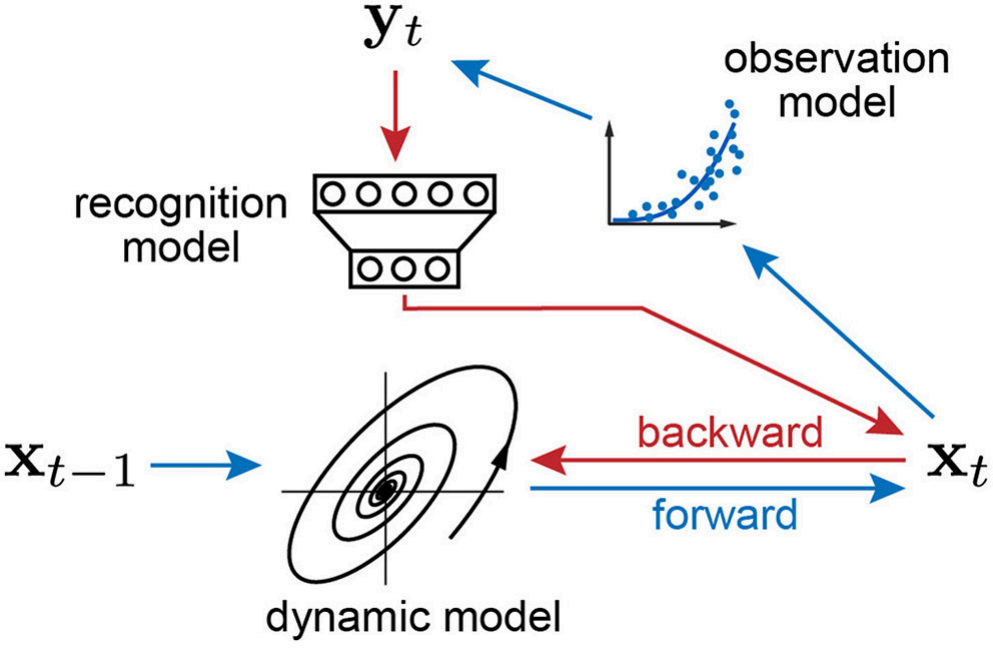
Schematics of variational joint filtering. Blue arrows indicates forward pass in which the previous latent state generates the new state through the state dynamics, and the new state transforms into the observation through the observation model. Red arrows indicate backward pass in which the recognition model recovers the current latent state from the observation. The three components, observation model, recognition model, and dynamical system, are updated by backpropagation.

The schematics of the proposed inference algorithm is summarized by two passes in Figure 1. In the forward pass, the previous latent state generates the new state through the dynamic model, and the new state transforms into the observation through the observation model. In the backward pass, the recognition model recovers the current latent state from the observation, and the observation model, recognition model, and dynamic model are updated by backpropagation.

Algorithm 1 is an overview of the recursive estimation algorithm. Denoting the set of all parameters by **Θ** of the observation model, recognition model and dynamic models, the objective function in Equation (6) is differentiable w.r.t. **Θ**, and we thus employ empirical Bayes and optimize it through stochastic gradient ascent (using Adam, Kingma and Ba, 2014). We outline the algorithm for a single vector time series, but we can filter multiple time series with a common state space model simultaneously, in which case the gradients are averaged across the instantiations. Note that this algorithm has *constant time and space complexity* per time step.

**Algorithm 1:** Variational Joint Filtering (single step).

**procedure** VJF(**y**_*t*_, **u**_*t*−1_, **μ**_*t*−1_, **s**_*t*−1_, **Θ**)
$\epsilon_{t}\leftarrow N\left(0,\text{I}\right),\epsilon_{t-1}\leftarrow N\left(0,\text{I}\right)$ ⊳ Draw random samples
[**μ**_*t*_, **s**_*t*_]: = **h**(**y**_*t*_, **u**_*t*−1_, **μ**_*t*−1_, **s**_*t*−1_) ⊳ State estimation
${\tilde{\text{x}}}_{t}: ={\text{μ}}_{t}+{\text{s}}_{t}^{1/2}\epsilon_{t}$
${\tilde{\text{x}}}_{t-1}: ={\text{μ}}_{t-1}+{\text{s}}_{t-1}^{1/2}\epsilon_{t-1}$
Update **Θ** with $\nabla_{\text{Θ}}\hat{L}\left(\text{Θ};{\text{y}}_{t},{\tilde{\text{x}}}_{t},{\tilde{\text{x}}}_{t-1},{\text{u}}_{t-1}\right)$ ⊳ Model update
**return μ**_*t*_, **s**_*t*_ and **Θ**
**end procedure**

In practice, the measurements **y**_*t*_ and input u_*t*_ are sampled at a regular interval. Algorithm 1 is called after every such observation event, which will return the state estimate along with the parameters and the dynamical system. One can visualize these for real-time for monitoring, and/or have it streamed to another system for further automated processing (e.g., detect anomalies and raise an alarm or deliver feedback controls).

## 3. Application to Latent Neural Dynamics

Our primary applied aim is real-time neural interfaces where a population of neurons are recorded while a low-dimensional stimulation is delivered (Newman et al., 2015; El Hady, 2016; Hocker and Park, 2019). State-space modeling of such neural time series have been successful in describing population dynamics (Macke et al., 2011; Zhao and Park, 2017). Moreover, models of neural computation are often described as dynamical systems (Hopfield, 1982; Dayan and Abbott, 2001; Barak et al., 2013). For example, attractor dynamics where the convergence to one of the attractors represents the result of computation (Wang, 2002; Nassar et al., 2019). Here, we propose a parameterization and tools for visualization of the model suitable for studying neural dynamics and building neural interfaces (Zhao and Park, 2016). In this section, we provide methodological details for the results presented in the next section.

### 3.1. Parameterization of the State Space Model

Having in mind high-temporal resolution neural spike trains where each time bin has at most one action potential per channel, we describe the case for point process observation. Our method, however, generalizes to arbitrary observation likelihoods that are appropriate for other modalities, including calcium imaging or local field potentials. The observed point process time series **y**_*t*_ is a stream of sparse binary vectors. All experiments of point process observation were binned finely so that the time bins contain one event each at most in this study.

Our observation model, Equation (7), assumes that the observation vector **y**_*t*_ is sampled from a probability distribution *P* determined by the latent state **x**_*t*_ though a linear-non-linear map possibly together with extra parameters at each time *t*,

(7) $$\begin{array}{l} {\text{y}}_{t}\sim P\left(g\left(\text{C}{\text{x}}_{t}+\text{b}\right)\right) \end{array}$$

where *g*:ℝ → ℝ is a point-wise map. We use the canonical link *g*(·) = exp(·) for Poisson likelihood and identity for Gaussian likelihood in this study. Note that this observation model is not identifiable since $\text{C}{\text{x}}_{t}=\left(\text{C}\text{R}\right)\left({\text{R}}^{-1}{\text{x}}_{t}\right)$ where **R** is an arbitrary invertible matrix. We normalize the loading matrix **C** in each iteration. It is straightforward to include more additive exogenous variables, a history filter for refractory period, coupling between processes, and stimulation artifacts (Truccolo et al., 2005; Pillow et al., 2008).

For state dynamic model, we propose using a specific additive parameterization with state transition function and input interaction as a special case of Equation (2),

(8a) $$\begin{array}{l} {\text{x}}_{t+1}={\text{x}}_{t}+f\left({\text{x}}_{t}\right)+{\text{B}}_{t}{\text{u}}_{t}+{\text{ϵ}}_{t+1} \end{array}$$

(8b) $$\begin{array}{l} f\left({\text{x}}_{t}\right)=\text{W}\text{ϕ}\left({\text{x}}_{t}\right) \end{array}$$

(8c) $$\begin{array}{l} {\text{x}}_{0},{\text{ϵ}}_{t}\sim N\left(0,\sigma^{2}\text{I}\right) \end{array}$$

where **ϕ**(·) is a vector of *r* continuous basis functions, i.e., $\text{ϕ}\left(\cdot \right)=(\phi_{1}\left(\cdot \right),\ldots ,\phi_{r}\left(\cdot \right){)}^{⊤}$, **W** is the weight matrix of the radial basis functions, and **B**_*t*_ is the interaction with the input **u**_*t*_. The interaction **B**_*t*_ can be globally linear, parameterized as a matrix independent from **x**_*t*_, or locally linear, parameterized as a matrix-valued function of **x**_*t*_ using also RBF networks. i.e., vec(**B**(**x**_*t*_)) = **W**_*B*_**ϕ**(**x**_*t*_) where **W**_*B*_ is the respective weight matrix. In this study, we used squared exponential radial basis functions (Roweis and Ghahramani, 2001; Sussillo and Barak, 2013; Frigola et al., 2014; Zhao and Park, 2016),

(9) $$\begin{array}{l} \phi_{i}\left(\text{x}\right)=\exp\left(-\frac{1}{2}\gamma_{i}∥\text{x}-{\text{c}}_{i}{∥}_{2}^{2}\right) \end{array}$$

with centers **c**_*i*_ and corresponding inverse squared kernel width γ_*i*_. Though the dynamics can be modeled by other universal approximators such as perception and RNN, we chose the radial basis function network for the reasons of non-wild extrapolation (zero velocity when the state is far away from data) and fast computation.

The time complexity of our algorithm is O(mpr+n(m+p+q)), where *n, m, p, q, r* denote the dimensions of observation, latent space, input, the numbers of hidden units, and radial basis functions for this specific parameterization. Practically to achieve realistic computation time for real-time applications in neuroscience, the number of radial basis functions and hidden units are constrained by the requirement. Note that the time complexity does not grow with time that enable efficient online inference. If we compare this to an efficient offline algorithm such as PLDS (Macke et al., 2011) run repeatedly for every new observation (“online mode”), its time complexity is $O\left(t\cdot \left(m^{3}+mn\right)\right)$ per time step at time *t*, which increases as time passes, making it impractical for real-time application.

### 3.2. Phase Portrait Analysis

Phase portrait displays key qualitative features of dynamics, and, with a little bit of training, it provides a visual means to interpreting dynamical systems. The law that governs neural population dynamics captured in the inferred function *f*(**x**) directly represents the velocity field of an underlying smooth dynamics (1a) in the absence of input (Roweis and Ghahramani, 2001; Zhao and Park, 2016). In the next section, we visualize the estimated dynamics as phase portrait which consists of the vector field, example trajectories, and estimated dynamical features (namely fixed points) (Strogatz, 2000). We can numerically identify candidate fixed points x^*^ that satisfy *f*(x^*^) ≈ 0. For the synthetic experiments, we performed an affine transformation to orient the phase portrait to match the canonical equations in the main text when the simulation is done through the proposed observation model if the observation model is unknown and estimated.

### 3.3. Prediction

For state space models, we can predict both future latent trajectory and future observations. The *s*-step ahead prediction can be sampled from the predictive distributions:

(10a) $$\begin{array}{l} p\left({\text{x}}_{t+1:t+s}|{\text{y}}_{\leq t}\right)={𝔼}_{q\left({\text{x}}_{t}\right)}\left[p\left({\text{x}}_{t+1:t+s}|{\text{x}}_{t}\right)\right] \end{array}$$

(10b) $$\begin{array}{l} p\left({\text{y}}_{t+1:t+s}|{\text{y}}_{\leq t}\right)={𝔼}_{p\left({\text{x}}_{t+1:t+s}|{\text{y}}_{\leq t}\right)}\left[p\left({\text{y}}_{t+1:t+s}|{\text{x}}_{t+1:t+s}\right)\right] \end{array}$$

given estimated parameters by current time *t* without seeing the data **y**_*t*+1:*t*+*s*_ during these steps. In the figures of experiments, we plot the mean of the predictive distribution as trajectories.

## 4. Experiments on Theoretical Models of Neural Computation

We demonstrate our method on a range of non-linear dynamical systems relevant to neuroscience. Many theoretical models have been proposed in neuroscience to represent different schemes of computation. For the purpose of interpretable visualization, we choose to simulate from two- or three-dimensional dynamical systems. We apply the proposed method to four such low-dimensional models: a ring attractor model as a model of internal head direction representation, a non-linear oscillator as a model of rhythmic population-wide activity, a biophysically realistic cortical network model for a visual discrimination experiment, and a chaotic attractor.

In the synthetic experiments, we first simulated state trajectories by respective differential equations, and we then generated either Gaussian or point process observations (to mimic spikes) via Equation (7) with corresponding distributions. The parameters **C** and **b** were randomly drawn, and they were constrained to keep firing rate < 60 Hz on average for realistic spiking behavior. All observations are spatially 200-dimensional unless otherwise mentioned. We refer to their conventional formulations under different coordinate systems, but our simulations and inferences are all done in Cartesian coordinates. Note that we focus on online learning in this study and always train our model with streaming data, even while comparing with offline methods.

The approximate posterior distribution is defined recursively in Equation (5) as diagonal Gaussian with mean and variance determined by corresponding observation, input, and previous step via a recurrent neural network. We used a one-hidden-layer MLP in this study. Typically, the state noise variance σ^2^ is unknown and has to be estimated from data. To be consistent with Equation (8c), we set the starting value of σ^2^ to be 1, and hence **μ**_0_ = **0**, **s**_0_ = **I**. We initialize the loading matrix **C** by factor analysis, and column-wisely normalize it by ℓ_2_ norm every iteration to keep the system identifiable.

### 4.1. Ring Attractor

Continuous attractors are often used as models for neural representation of continuous variables (Mante et al., 2013; Sussillo and Barak, 2013). For example, a bump attractor network with ring topology is proposed as the dynamics underlying the persistently active set of neurons that are tuned for the angle of the animal's head direction (Peyrache et al., 2015). Here we use the following two-variable reduction of the ring attractor system. First, we study the following two-variable ring attractor system:

(11) $$\begin{array}{l} \begin{array}{l} \tau_{r}ṙ=r_{0}-r\\ \tau_{\varphi }\dot{\varphi }=I \end{array} \end{array}$$

where φ represents the direction driven by input *I*, and *r* is the radial component representing an internal circular variable, such as head direction. We simulated 100 trajectories (1,000 steps) with step size Δ*t* = 0.1, *r*_0_ = 1, τ_*r*_ = 1, and τ_φ_ = 1 with Gaussian state noise (std = 0.005) added each step. Though the ring attractor is defined in polar coordinate system, we transformed it into Cartesian system for simulation and training. In simulation we used strong input (tangent drift) to keep the trajectories flowing around the ring clockwise or counterclockwise. The point process observations were generated by passing the states through a linear-non-linear map (Equation 7) and sampling from a Poisson distribution. We streamed the observations into the proposed algorithm that consists of point process likelihood, a dynamic model with 20 radial basis functions and locally linear input interaction in Equation (2), and a recognition MLP with 100 hidden units.

**Figure 2:**
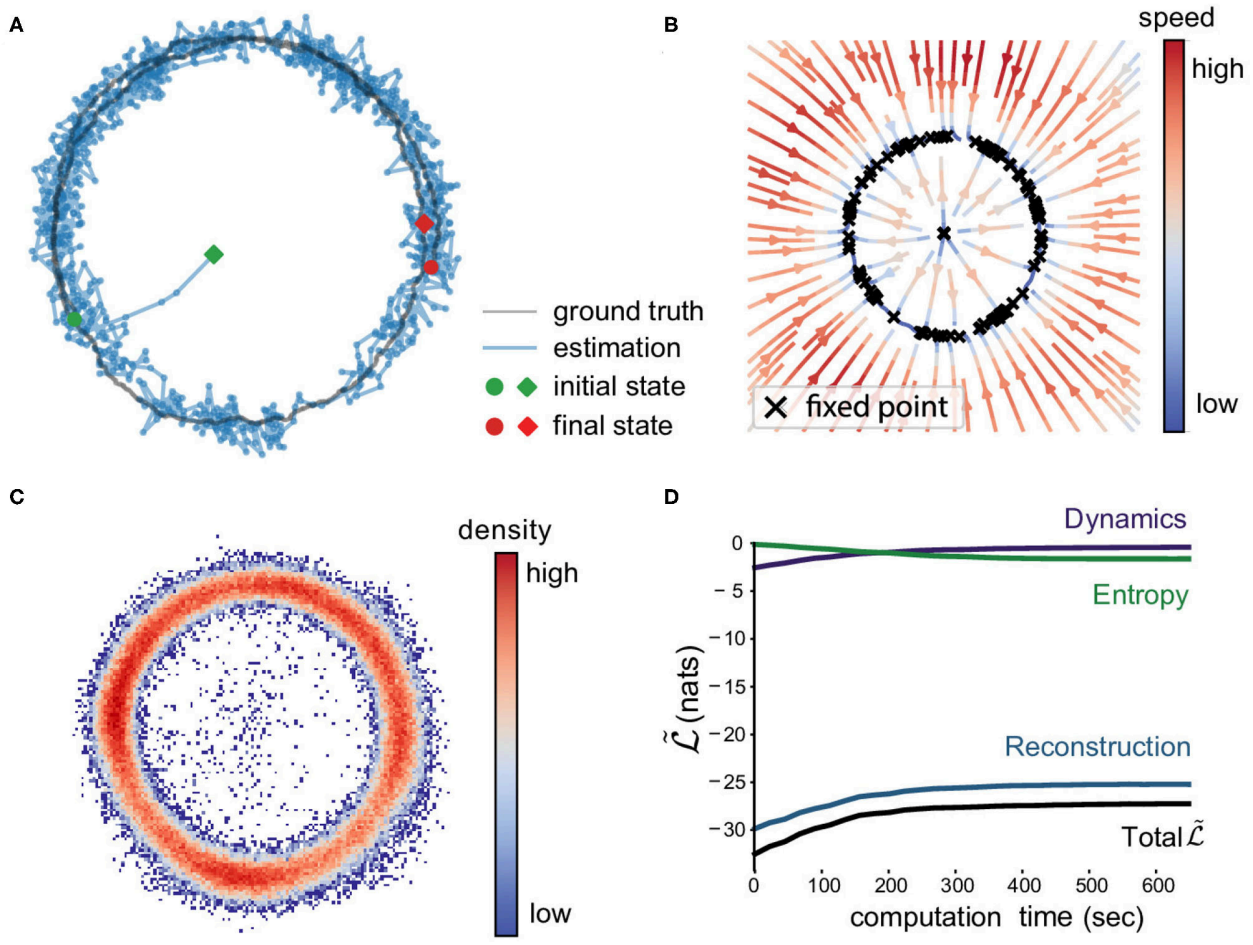
Ring attractor model. **(A)** One latent trajectory (black) in the training set and the corresponding filtered mean μ_*t*_ (blue). **(B)** Velocity field reconstructed from the trained proposed model. The colored streamlines indicate the speed and the directions. The black crosses are candidate fixed points obtained from inferred dynamics. Note the collection of fixed points around the ring shape. The central fixed point is unstable. **(C)** Density of the posterior means. The density of inferred means of all trajectories in the training set. The higher it is, the more confidence we have on the inferred dynamics where we have more data. **(D)** Convergence on the ring attractor. We display the three components of the objective lower bound: reconstruction log-likelihood, dynamics log-likelihood, entropy, and the lower bound itself from Equation (4). The average computation time per step is 1.1 ms (more than 900 data points per sec).

Figure 2A illustrates one latent trajectory (black) and its variational posterior mean (blue). These two trajectories start at green circle and diamond respectively and end at the red markers. The inference starts near the center (origin) that is relatively far from the true location because the initial posterior mean is set at zero. The final states are very close, which implies that the recognition model works well. Figure 2B shows the reconstructed velocity field by the model. We visualized the velocity as colored directional streamlines. We can see the velocity toward the ring attractor and the speed is smaller closer to the ring. The model also identifies a number of fixed points arranged around the ring attractor via numerical roots finding. Figure 2C shows the distribution of posterior means of all data points in the state space. We have more confidence of the inferred dynamical system in the denser area.

Figure 2D shows the three components of Equation (4) and the objective lower bound clearly, demonstrating the convergence of the algorithm. We can see each component reaches a plateau within 400 s. As the reconstruction and dynamics log-likelihoods increase, the recognition model and dynamical model are getting more accurate while the decreasing entropy indicates the increasing confidence (inverse posterior variance) on the inferred latent states. The average computation time of a joint estimation step is 1.1 ms (hardware specification: Intel Xeon E5-2680 2.50G Hz, 128GB RAM, no GPU).

### 4.2. Non-linear Oscillator

Dynamical systems have been a successful application in the biophysical models of single neuron in neuroscience. We used a relaxation oscillator, the FitzHugh-Nagumo (FHN) model (Izhikevich, 2007), which is a two-dimensional reduction of the Hodgkin-Huxley model with the following non-linear state dynamics

(12) $$\begin{array}{l} \begin{array}{l} \dot{v}=v\left(a-v\right)\left(v-1\right)-w+I,\\ ẇ=bv-cw, \end{array} \end{array}$$

where *v* is the membrane potential, *w* is a recovery variable, and *I* is the magnitude of stimulus current in modeling single neuron biophysics. This model was also used to model global brain state that fluctuates between two levels of excitability in anesthetized cortex (Curto et al., 2009). We use the following parameter values *a* = −0.1, *b* = 0.01, *c* = 0.02, and *I* = 0.1 to simulate 100 trajectories of 1,000 steps with step size 0.5 and Gaussian noise (std=0.002). At this regime, unlike the ring attractor, the spontaneous dynamics is a periodic oscillation, and the trajectory follows a limit cycle. The point process observations were also sampled via the observation model of the same parametric form as that of the ring attractor example. We used 20 radial basis functions for dynamic model and 100 hidden units for recognition model. While training the model, the input was clamped to zero, and the model was expected to learn the spontaneous oscillator.

**Figure 3:**
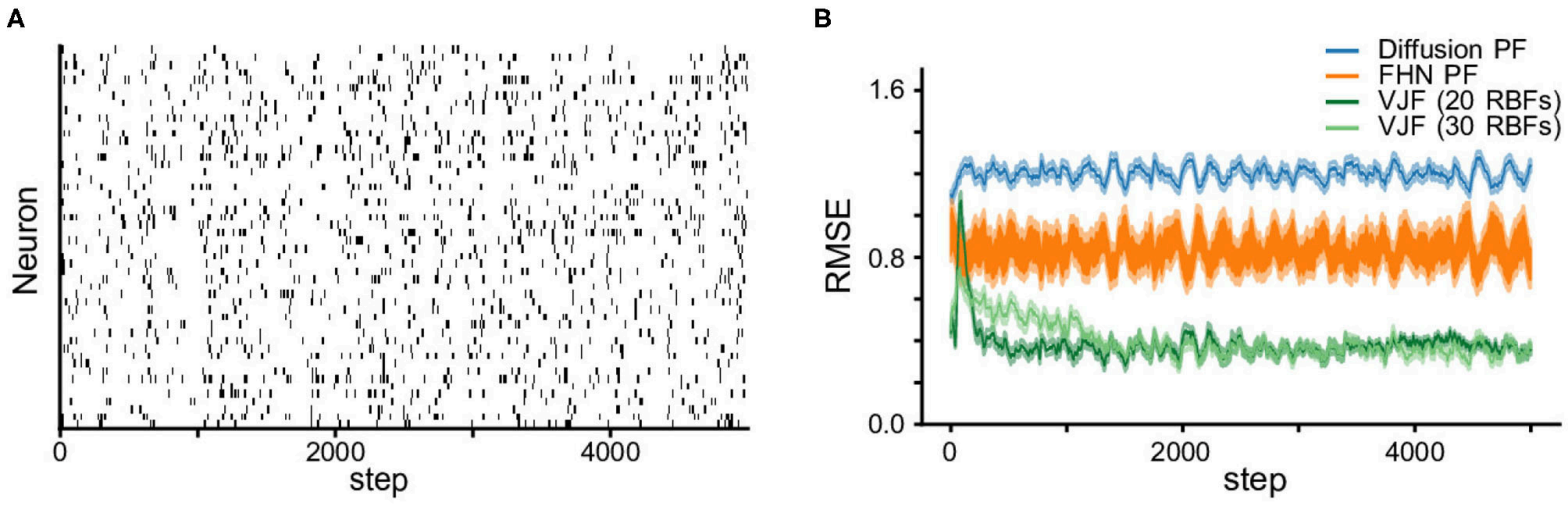
Non-linear oscillator (FitzHugh-Nagumo) observation and state estimation. **(A)** The synthetic spike train. **(B)** RMSEs (the shades are s.e.m.) of state estimation on the observation in **(A)**. We report the RMSE of estimated states among the proposed method VJF and two particle filters, one with diffusion dynamics and the other with the true FHN dynamics. Varying the number of RBFs did not substantially change the quality of the results.

We compare the state estimation with the standard particles filtering (PF), which are powerful online methods theoretically capable of producing arbitrarily accurate filtering distribution. We run two variants of the particle filter with different proposal distributions. One used diffusion as the proposal, i.e., **x**_*t*_ = **x**_*t*−1_ + ϵ_*t*_ where **x** is the vector of state variables *v* and *w*, and the other, a.k.a. bootstrap particle filter (Gordon et al., 1993), used the true dynamics in Equation (12). We provided the true parameters for the observation model and noise term to PF, which gives them an advantage. Both particle filters and VJF were run on 50 realizations of 5,000-step long observation series. Figure 3 shows the root mean squared deviations (RMSE) (mean and standard error over 50 realizations). It is expected that the bootstrap particle filter outperformed the diffusion particle filter since the former utilized the true dynamics. One can see the state estimation by VJF improved as learning carrying on and eventually outperformed both particle filters. Note that VJF had to learn the parameters of likelihood, dynamic model and recognition model during the run. We varied the number of RBFs (20 and 30), but the results are not substantially different.

**Figure 4:**
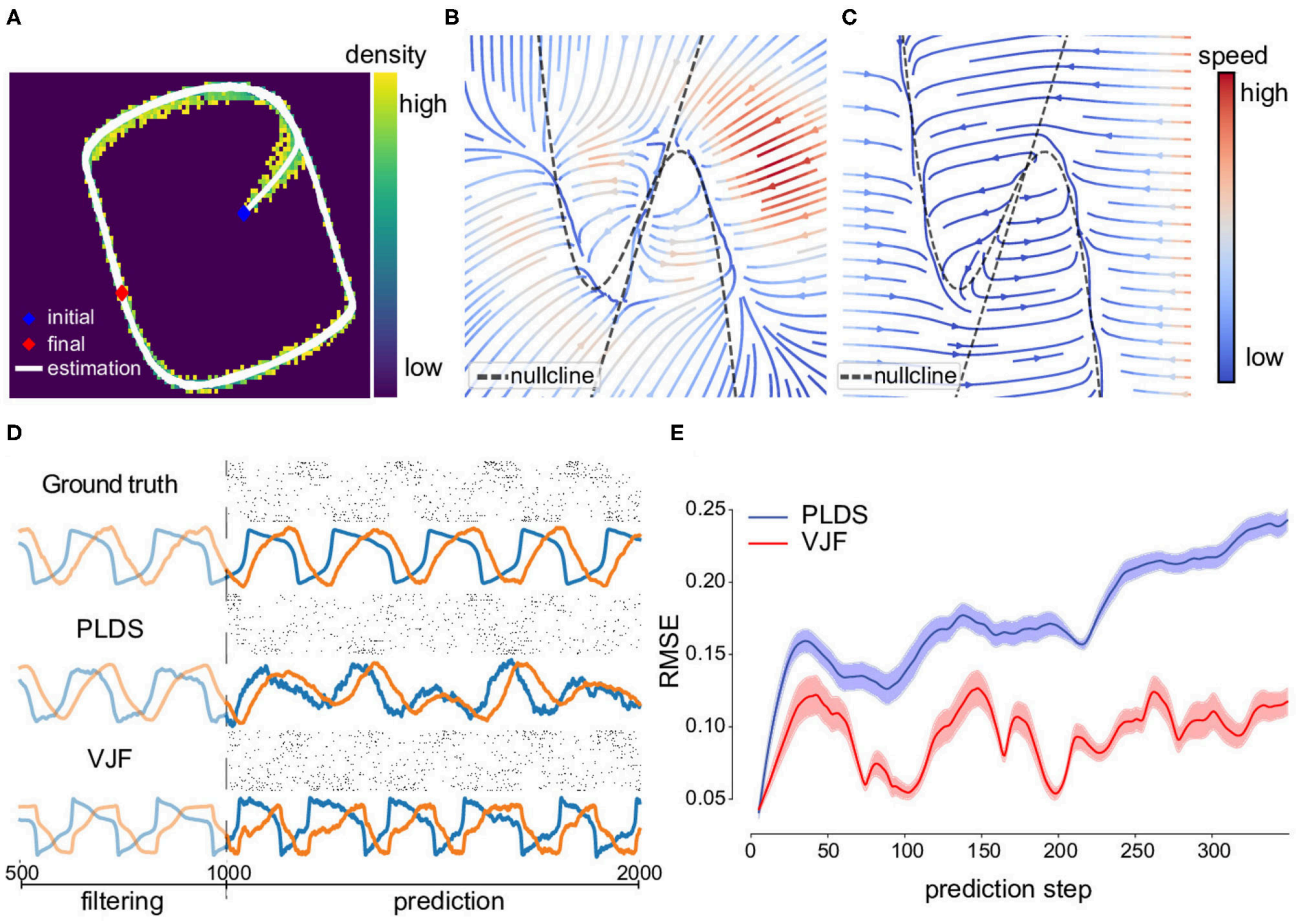
Non-linear oscillator (FitzHugh-Nagumo) dynamical system and prediction. **(A)** One inferred latent trajectory and the density of posterior means of all trajectories. Most of the inferred trajectory lies on the oscillation path. **(B)** Velocity field reconstructed by the inferred dynamical system. **(C)** Velocity field of the true dynamical system. The dashed lines are two nullclines of the true model on which the gradients are zero so as the velocity. **(D)** 1,000-step prediction continuing the trajectory and sampled spike trains compared to ground truth from **(A)**. **(E)** Mean (solid line) and standard error (shade) of root mean square error of prediction of 2,000 trials. The prediction started at the same states for the true system and models. Note that PLDS fails to predict long term due to its linear dynamics assumption. A linear dynamical system without noise can only produce damped oscillations.

We also reconstructed the phase portrait (Figure 4B) comparing to the truth (Figure 4C). The two dashed lines are the theoretical nullclines of the true model on which the velocity of corresponding dimension is zero. The reconstructed field shows a low speed valley overlapping with the nullcline especially on the right half of the figure. There is an unstable fixed point at the intersection of the two nullclines. We can see the identified fixed point is close to the intersection. As most of the trajectories lie on the oscillation path (limit cycle) with merely few data points elsewhere, the inferred system shows the oscillation dynamics similar to the true system around the data region. The difference mostly happens in the region far from the trajectories because of the lack of data.

We ran a long-term prediction using VJF without seeing the future data **y**_*t*+1:*T*_ during these steps (*T* = 1,000 steps = 1 s) beginning at the final state of training data. The truth and prediction can be seen in Figure 4D. The upper row is the true latent trajectory and corresponding observations. The lower row is the filtered trajectory and prediction by the proposed method. The light-colored parts are the 500 steps of inference before prediction and the solid-colored parts are 1,000-step truth and prediction. We also show the sample observations from the trained observation model during the prediction period.

One of the popular latent process modeling tools for point process observation that can make prediction is the Poisson Linear Dynamical System (PLDS) (Macke et al., 2011) which assumes latent linear dynamics. We compared PLDS fit with EM on its long-term prediction on both the states and spike trains (Figure 4). This demonstrates the non-linear dynamical model outperforming the linear model even in the unfair online setting.

To compare to the methods with non-linear dynamical models, we also run latent factor analysis via dynamical systems (LFADS) (Pandarinath et al., 2018) offline using the same data. LFADS implements its dynamical model with the gated recurrent unit (GRU) (Cho et al., 2014) that requires high dimensions. For this two-dimensional system, we tried different GRU dimensionalities. We made minimal changes to its recommended setting, including only the generator dimensionality, batch, and no controller. The result shows that LFADS requires much higher dimension than the true system to capture the oscillation (Figure S1). (The figure of its inferred trajectories is shown in the supplement.) We report the fitted log-likelihood per time bin as −0.1274, −0.1272, and −0.1193 for 2D, 20D, and 50D GRU, respectively. In comparison, the log-likelihood of the proposed approach is −0.1142 with a two-dimensional dynamical model (higher the better).

### 4.3. Fixed Point Attractor for Decision-Making

**Figure 5:**
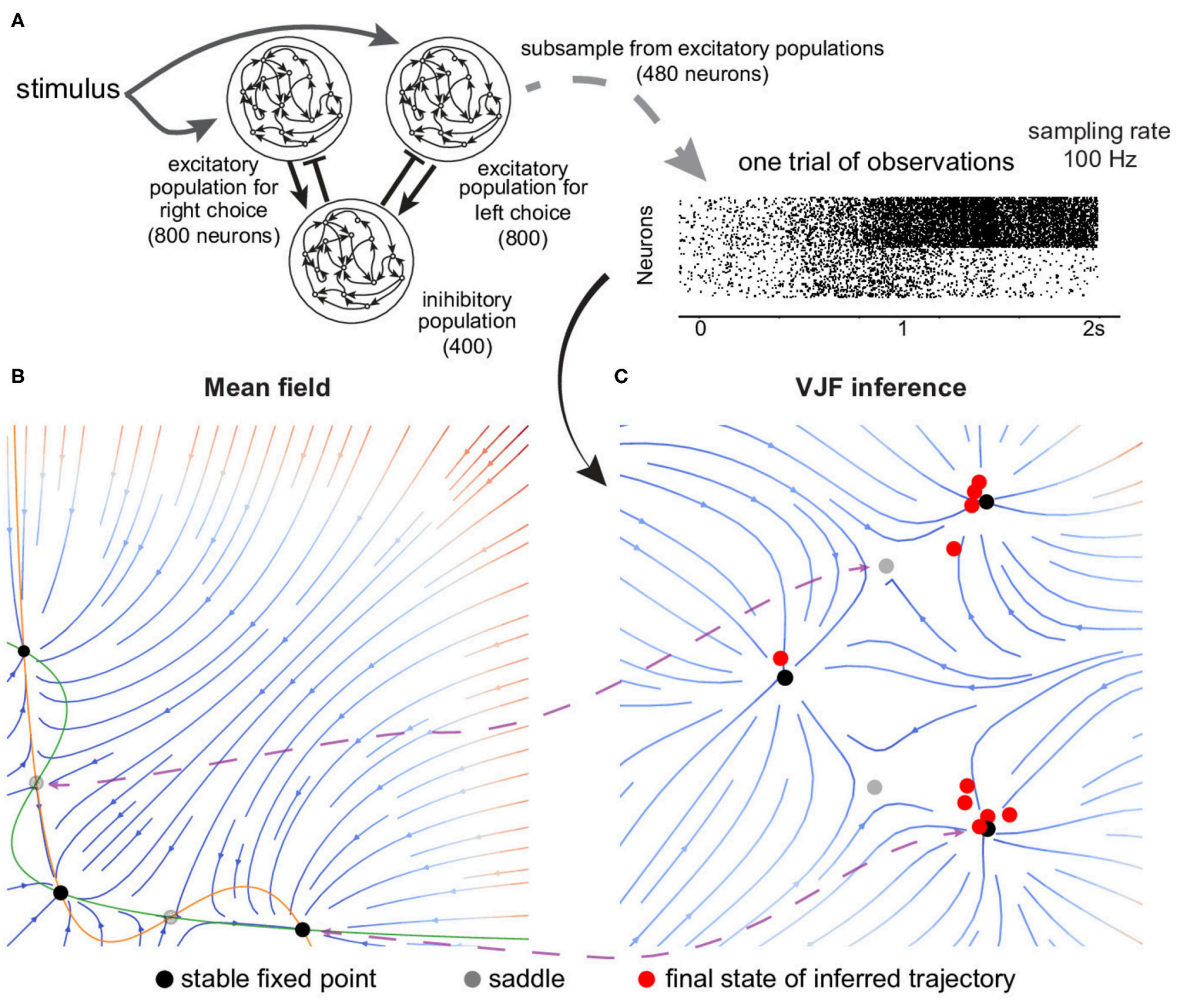
Fixed point attractor for decision-making. **(A)** Schematics of the neural network. There are two excitatory populations that are wired with slow recurrent excitation and feedback inhibition to produce attractor dynamics. The simulation was organized into decision-making trials. Each trial begins with a 0.5 s period of spontaneous activity, and the input is then given to the two excitatory populations for 1.5 s. We subsampled 480 selective neurons out of 1,600 excitatory neurons from the simulation to be observed by our algorithm. **(B)** Mean field reduction of the network. Theoretical work has shown that the collective population dynamics can be reduced to two dimensions (Wong and Wang, 2006). **(C)** VJF inferred dynamical model. The red dots are the inferred final states of zero-coherent trials. The black dots are fixed points (the solid are stable and the gray are unstable). Although the absolute arrangement is dissimilar, the topology and relation of the five identified fixed points show correspondence (indicated by purple lines).

Perceptual decision-making paradigm is a well-established cognitive task where typically a low-dimensional decision variable needs to be integrated over time, and subjects are close to optimal in their performance. To understand how the brain implements such neural computation, many competing theories have been proposed (Wang, 2002; Wong and Wang, 2006; Ganguli et al., 2008; Barak et al., 2013; Mante et al., 2013). We test our method on a simulated biophysically realistic cortical network model for a visual discrimination experiment (Wang, 2002). In the model, there are two excitatory subpopulations that are wired with slow recurrent excitation and feedback inhibition to produce attractor dynamics with two stable fixed points (Figure 5A). Each fixed point represents the final perceptual decision, and the network dynamics amplify the difference between conflicting inputs and eventually generates a binary choice.

Unlike former examples that use a linear-non-linear map of latent states, the point process observations (spikes) of this experiment were directly sampled from the spiking neural network^1^(1 ms binwidth) that was governed by its own high-dimensional intrinsic dynamics. It is filling the gap between fully specified state space models and real neuron populations.

We subsampled 480 selective neurons out of 1,600 excitatory neurons from the simulation to be observed by our algorithm. The simulated data is organized into decision-making trials where each trial lasts for 2 s and with different strength of visual evidence, controlled by “coherence.” Our method with 20 radial basis functions learned the dynamics from 140 training trials (20 per coherence level *c*, *c* = −1, −0.2, −0.1, 0, 0.1, 0.2, *and*1).

Figure 5C shows the velocity field at zero coherence stimulus as colored streamlines. Note that our approach did not have prior knowledge of the network dynamics as the mean-field reduction (Wong and Wang, 2006) in Figure 5B. Although the absolute arrangement is dissimilar, the topology and relation of the five identified fixed points show correspondence with the mean-field reduction. The inference was completely data-driven (partial observation of spike trains), while the mean-field method required knowing the true dynamical model of the network and careful approximation by Wong and Wang (2006). We showed that our method can provide a qualitatively similar result to the theoretical work, which reduces the dimensionality and complexity of the original network.

### 4.4. Chaotic Dynamics

Chaotic dynamics (or edge-of-chaos) have been postulated to support asynchronous states in the cortex and neural computation over time by generating rich temporal patterns (Maass et al., 2002; Laje and Buonomano, 2013). We consider the three-dimensional standard Lorenz attractor as an example chaotic system to demonstrate the flexibility of our method. We simulated 216 latent trajectories from:

(13) $$\begin{array}{l} ẋ=10\left(y-x\right),\;ẏ=x\left(28-z\right)-y,\;ż=xy-\frac{8}{3}z. \end{array}$$

**Figure 6:**
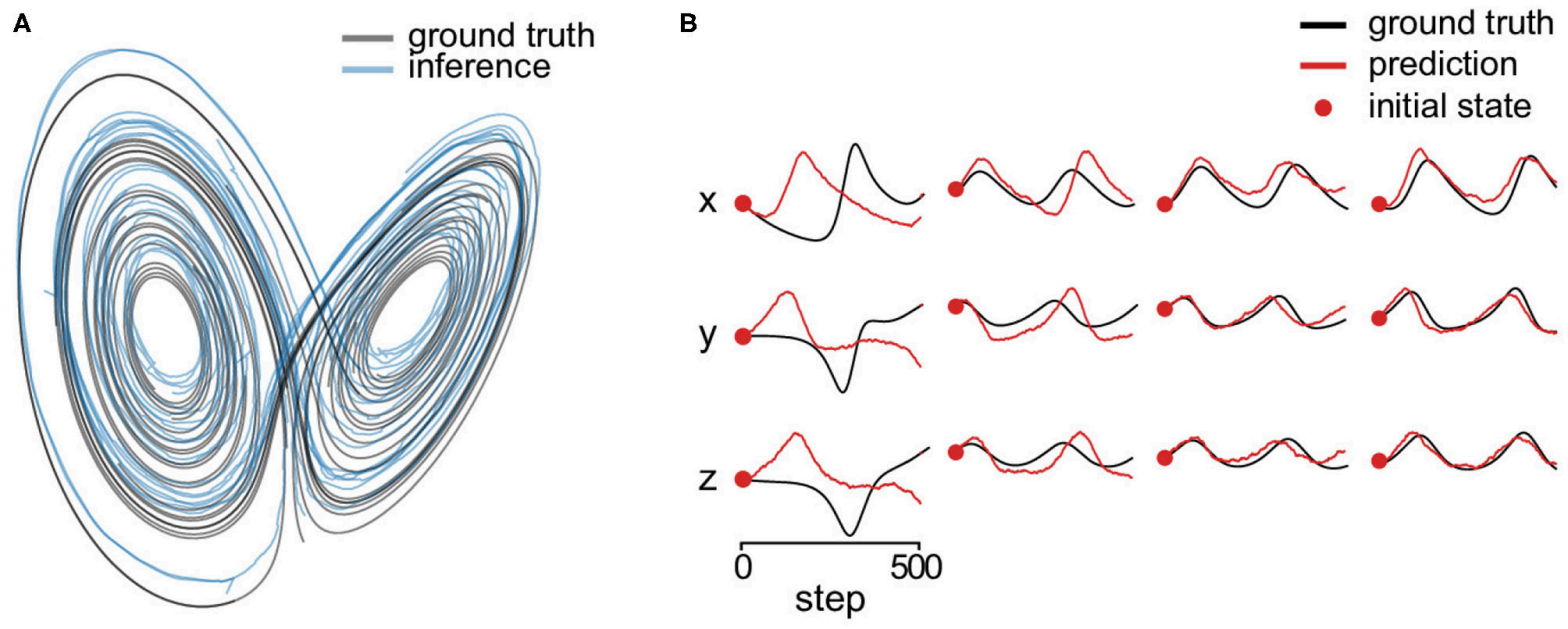
Lorenz attractor. **(A)** Estimated state trajectory (blue) and the ground truth (black) in three dimensions. **(B)** We predict 500 steps of future latent states (without knowing the respective observations) starting from four different initial states (red dots) using the inferred dynamical system. The red lines are the prediction and the black lines are the corresponding ground truth state.

The each coordinate of the initial states are on the uniform grid of 6 values in [−50, 50] inclusively, of which the combination results in 216 unique states. We discarded the first 500 transient steps of each trajectory and then use the following 1,000 steps. We generated 200-dimensional Gaussian observations driven by the trajectories. Figure 6A shows estimated latent trajectory and the ground truth. One can see that the estimation lies in a similar manifold. In addition, we predicted 500 steps of future latent states without knowing the respective observations. Figure 6B shows four predicted trajectories starting from different initial states. One can see that the inferred system could generate qualitatively similar trajectory at most initial states but not perfectly for the true system is chaotic.

### 4.5. Non-stationary System

**Figure 7:**
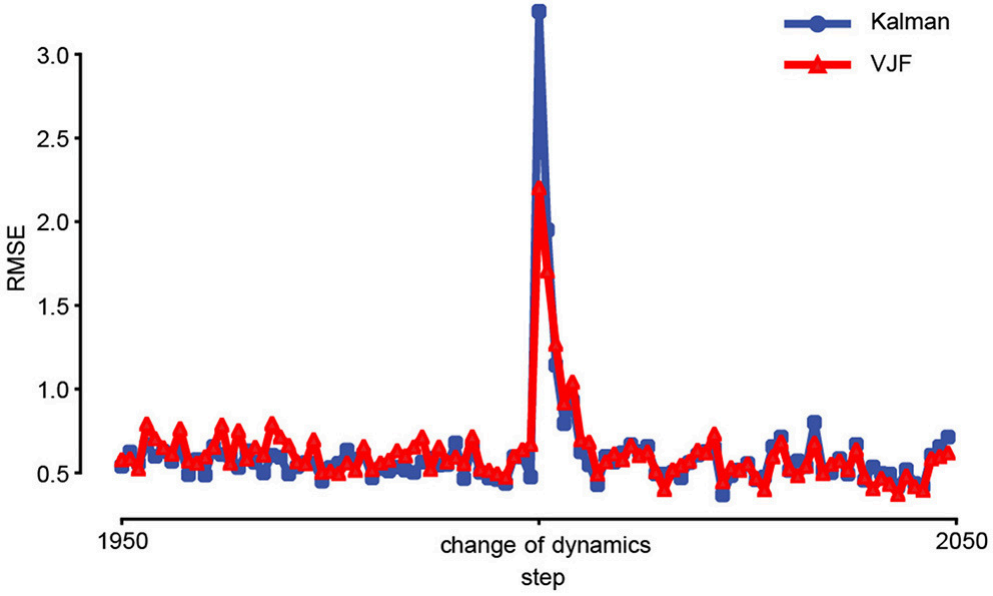
Prediction of non-stationary dynamical system. The colored curves (blue: EKF, red: VJF) are the mean RMSEs of one-step-ahead prediction of non-stationary system during online learning (50 trials). The linear system was changed, and the state was perturbed at the 2000th step (center). The lines are average RMSEs. Both online algorithms quickly learned the change after a few steps.

Another feature of our method is that its state dynamics estimate never stops. As a result, the algorithm is adaptive, and can potentially track slowly varying (non-stationary) latent dynamics. To test this feature, we compared a dual EKF and the proposed approach on non-stationary linear dynamical system. A spiral-in linear system was suddenly changed from clockwise to counterclockwise at the 2000th step, and the latent state was perturbed (Figure 7). To adapt EKF, we used Gaussian observations that were generated through linear map from a two-dimensional state to 200-dimensional observation with additive noise (N(0,0.5)). To focus on the dynamics, we fixed all the parameters except the transition matrix for both methods, while our approach still must learn the recognition model in addition. Figure 7 shows that our approach achieved better online performance as dual EKF in this experiment.

## 5. Real Neurophysiological Application

**Figure 8:**
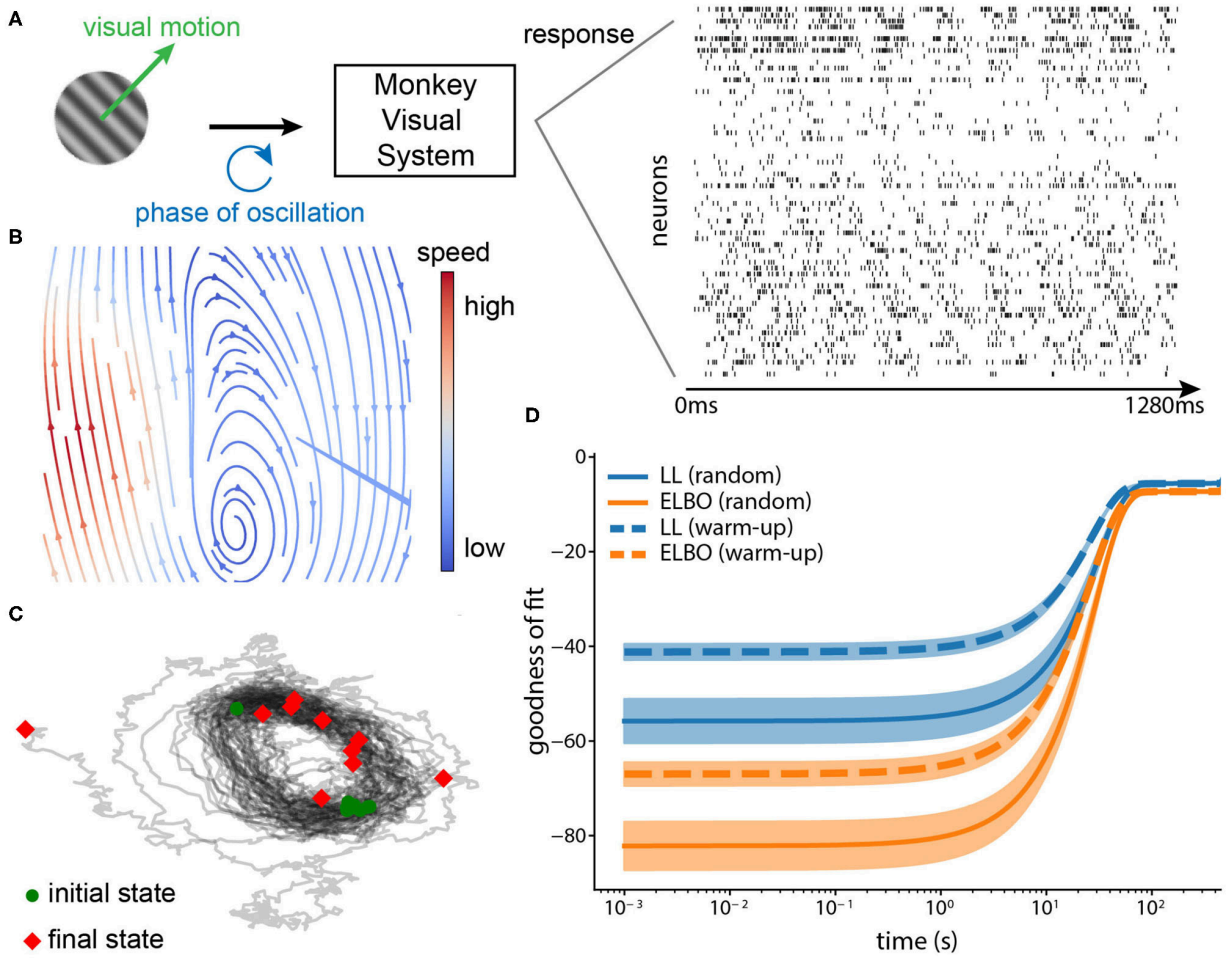
**(A)** Neurophysiological experiment. Drifting gratings were shown to the monkey (on the left). The neural spike trains (63 neurons, 1,280 ms) from area V1 during the motion onset were recorded (on the right). Each row is one neuron and the binwidth is 1 ms. The phase of the oscillation forms a circular variable. **(B)** Phase portrait of the inferred dynamical system (arrows: direction, blue: low speed, and red: high speed). The flow shows that the inferred system forms an oscillator. **(C)** Trajectories simulated from the inferred dynamical system. We simulated 10 state trajectories using the inferred system with random initial states (1,000 steps each, black lines: trajectories, green circles: initial states, red diamonds: final states). The trajectories also confirm that the inferred system captured the oscillation underlying the data. **(D)** Convergence of the online method in terms of its goodness-of-fit. We calculated two goodness-of-fit measures (mean ± standard deviation, 10 repetitions), log-likelihood (LL) and ELBO for two strategies of initializing the observation model, warm-up and random initialization. Warm-up indicates that we initialized the observation model using dimensionality reduction methods before VJF; Random initialization indicates that the parameters of observation model were randomly drawn and learned completely by VJF.

We applied the proposed method to a large-scale recording to validate that it picks up meaningful dynamics. The dataset (Graf et al., 2011) consists of 148 simultaneously recorded single units from the primary cortex (V1) while directional drifting gratings were presented to an anesthetized monkey for around 1.3s per trial (Figure 8A). We used the spike trains from 63 well-tuned units. The spike times were binned with a 1ms window (max 1 spike per bin). There is one continuous circular variable in the stimuli space: temporal phase of oscillation induced by the drifting gratings.

A partial warm-up helps with the training. We chose a good initialization for the observation model, specifically the loading matrix and bias. There are 72 motion directions in total, each repeated 50 trials. We used the trials corresponding to 0 deg direction to initialize the observation model with dimensionality reduction methods, such as variational latent Gaussian processes Zhao and Park (2017), and then trained VJF with a two-dimensional dynamic model fully online on the trials corresponding to 180 deg direction that it had not seen before. Since we do not have long enough continuously-recorded trials, we concatenated the trials (equivalent to 500 s) as if they were continuously recorded to mimic an online setting. As expected, Figures 8B,C shows the inferred dynamical system is able to implement the oscillation. The two goodness of fit measures (log-likelihood and ELBO) in Figure 8D shows that our method benefits from but does not necessarily require such a warm-up. The model with warm-up initialization had better starting goodness of fit than the random initialized model, but the random initialized model eventually achieved similar goodness of fit with adequate amount of data.

## 6. Discussion

Neurotechnologies for recording the activity of large neural populations during meaningful behavior provide exciting opportunities for investigating the neural computations that underlie perception, cognition, and decision-making. However, the datasets provided by these technologies currently require sophisticated offline analyses that slow down the scientific cycle of experiment, data analysis, hypothesis generation, and further experiment. Moreover, in closed-loop neurophysiological setting, real-time adaptive algorithms are extremely valuable (Jordan and Park, 2020).

To fulfill this demand, we proposed an online algorithm for recursive variational Bayesian inference that simultaneously performs system identification and state filtering under the framework of state space modeling, in hope that it can greatly impact neuroscience research and biomedical engineering. There is no other method capable of all features, hence we compared several methods in different measures, often giving them the advantage. We showed that our proposed method consistently outperforms the state-of-the-art methods.

Using the language of dynamical systems, we interpret the target system not via model parameters but via dynamical features: fixed points, limit cycles, strange attractors, bifurcations, and so on. In our current approach, this interpretation heavily relies on visual inspection of the qualitative non-linear dynamical system features. In contrast, most popular state space models assume linear dynamics (Ho and Lee, 1964; Katayama, 2005; Macke et al., 2011), which is appropriate for smoothing latent states is but not expressive enough to recover the underlying vector field. Recently the Koopman theory that allows representation of general non-linear dynamics as linear operators in infinite dimensional spaces (Koopman, 1931) has gained renewed interest in modeling non-linear dynamics. Although elegant in theory, in practice, however, the Koopman operators need to be truncated to a finite dimensional space with linear dynamics (Brunton et al., 2016). We note that the resulting linear models do not allow for topological features such as multiple isolated fixed points, non-linear continuous attractors, stable limit cycles—features critical for non-trivial neural computation.

Our algorithm is highly flexible and general, allowing for a wide range of observation models (likelihoods) and dynamic models, is computationally tractable, and produces interpretable visualizations of complex collective network dynamics. Our key assumption is that the dynamics consists of a continuous and slow flow, which enable us to parameterize the velocity field directly. This assumption reduces the complexity of the non-linear function approximation; it is therefore easy to identify the fixed/slow points. We specifically chose the radial basis function network to model the dynamics for our experiments, which regularizes and encourages the dynamics to occupy a finite phase volume around the origin.

Our method has several hyperparameters. In the experiments, the differentiable hyperparameters were learnt via gradient descent while the selection of the other hyperparameters were made simple. In general, our method was robust; Perturbing the number of RBFs did not produce qualitatively different results (Figure 3). Liu et al. (2009) discussed growing radial basis function network adaptively, which could be incorporated into our method to enable online tuning of the number of RBFs. The depth and width of neural networks were chosen empirically to improve the interpretability of resulting dynamical systems, but tuning did not result in large changes in the results.

This work opens many avenues for future work. One direction is to apply this model to large-scale neural recording from a behaving animal. We hope that further development would enable on-the-fly analysis of high-dimensional neural spike train during electrophysiological experiments. Clinically, a non-linear state space model provides a basis for non-linear feedback control as a potential treatment for neurological diseases that arise from diseased dynamical states.

## Data Availability Statement

The datasets generated for this study are available on request to the corresponding author.

## Author Contributions

All authors developed the methods. All authors conducted all simulations and analyses and created all figures in the manuscript. All authors wrote the manuscript.

## Conflict of Interest

The authors declare that the research was conducted in the absence of any commercial or financial relationships that could be construed as a potential conflict of interest.

## Footnotes

^1^The detail of the spiking neural network can be found in Wang (2002), and the code can be found at https://github.com/xjwanglab/book/tree/master/wang2002.
