# Supplementary material for: Variational Online Learning of Neural Dynamics
Source: Front Comput Neurosci. 2020 Oct 14;14:71. doi: 10.3389/fncom.2020.00071 (PMC7591751; doi:10.3389/fncom.2020.00071)
Supplement: Supplementary file 1 [file Data_Sheet_1.PDF]

Supplementary Material

1 LFADS RESULT OF THE NONLINEAR OSCILLATOR

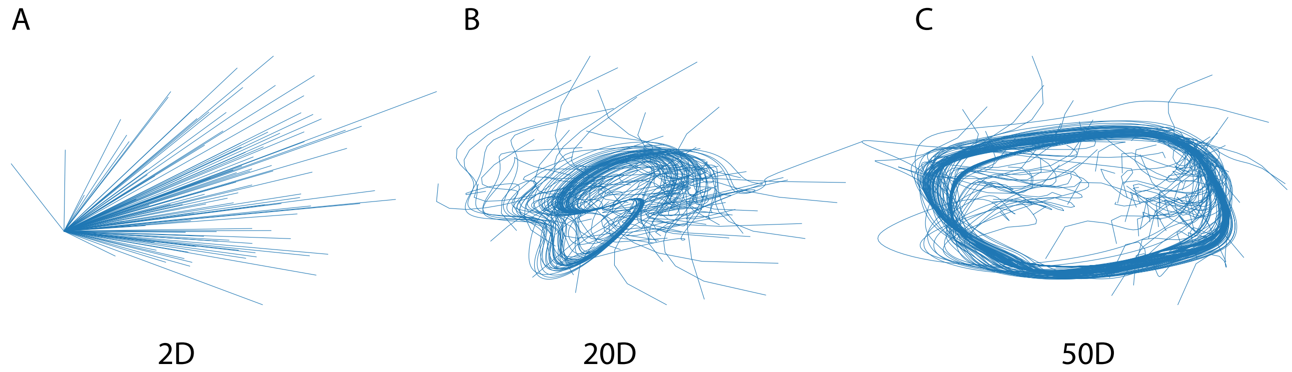

Figure S1. The first 2 PCs of inferred latent trajectories of FHN system by LFADS. We fit LFADS with 2D, 20D and 50D latent space. LFADS inherently requires much higher-dimensional latent space to recover the oscillation. We report the fitted log-likelihoods per time bin. On the contrary, the proposed approach gives a log-likelihood -0.1142.
